# Lnc-HSD17B11-1:1 Functions as a Competing Endogenous RNA to Promote Colorectal Cancer Progression by Sponging miR-338-3p to Upregulate MACC1

**DOI:** 10.3389/fgene.2020.00628

**Published:** 2020-06-12

**Authors:** Wei Zhang, Bo Wang, Quan Wang, Zhen Zhang, Zhanlong Shen, Yingjiang Ye, Kewei Jiang, Shan Wang

**Affiliations:** ^1^Department of Gastroenterological Surgery, Peking University People’s Hospital, Beijing, China; ^2^Laboratory of Surgical Oncology, Peking University People’s Hospital, Beijing, China; ^3^Beijing Key Laboratory of Colorectal Cancer Diagnosis and Treatment Research, Peking University People’s Hospital, Beijing, China

**Keywords:** colorectal cancer, lnc-HSD17B11-1:1, miR-338-3p, MACC1, competing endogenous RNA

## Abstract

**Background:**

Long non-coding RNAs (lncRNAs) play pivotal roles in various kinds of human diseases, especially in cancer. However, regulatory role, clinical significance and underlying mechanisms of lncRNAs in colorectal cancer (CRC) liver metastasis still remain largely unknown. This study aimed to report a novel lncRNA, lnc-HSD17B-11:1, and its functional role in CRC progression.

**Materials and methods:**

Differentially expressed lnc-HSD17B11-1:1 was screened and identified from a lncRNA profile microarray. Quantitative real-time PCR was used to determine the expression levels and prognostic values of lncRNA in CRC cohorts. *In vitro* and *in vivo* functional experiments were performed to investigate the effects of lnc-HSD17B11-1:1 on tumor growth and metastasis in CRC. Mechanistically, Base Scope, bioinformatics analyses, dual luciferase reporter assay and RNA immunoprecipitation experiments were performed to confirm the association of lnc-HSD17B11-1:1 and miR-338-3p. Dual luciferase reporter assay, qRT-PCR and western blot analysis were performed to assess the relationships among lnc-HSD17B11-1:1, miR-338-3p, and MACC1.

**Results:**

Evidently up-regulation of lnc-HSD17B11-1:1 in CRC primary tissues was correlated with the depth of invasion (*p* = 0.043), clinical stage (*p* = 0.027), distant metastasis (*p* = 0.003) and poor prognosis of patients with CRC. lnc-HSD17B11-1:1 promoted CRC cell proliferation, mobility and invasion *in vitro* and *in vivo*. Mechanistic analysis revealed that lnc-HSD17B11-1:1 may act as a competing endogenous RNA (ceRNA) by acting as a sponge for miR-338-3p to upregulate the expression of MACC1.

**Conclusion:**

These findings suggest that lnc-HSD17B11-1:1 promotes CRC progression through lnc-HSD17B11-1:1/miR-338-3p/MACC1 axis and this might serve as a new diagnostic marker or target for treatment of CRC.

## Background

Colorectal cancer (CRC) is the third most common cancer and the fifth most common cause of cancer-related death in China (Dekker et al., 2019; Siegel et al., 2020). The most commonly used treatment strategy for the management of CRC includes surgical resection followed by adjuvant chemotherapy. Although the overall 5-year survival rate of CRC has been improved to 65%, CRC patients with distant metastasis, especially liver metastasis are considered not suitable for surgical treatment and therefore the 5-year survival rate in these is <10%(Landreau et al., 2015; Siegel et al., 2016)reflecting the poor treatment response in some patients. Therefore, it is necessary to identify effective therapeutic strategies to improve treatment and prognosis for patients diagnosed with CRC.

Long non-coding RNAs (lncRNAs, >200 nucleotides in length) are recently discovered as a novel class of genes with regulatory functions but lacked protein-coding ability. Several studies have identified and reported important roles of lncRNAs in varied cellular processes, including X chromosome inactivation, splicing, imprinting, epigenetic control, and gene transcription regulation (Butler et al., 2016; Kanduri, 2016; Yue et al., 2016). Moreover, the dysregulated expression of lncRNAs is present in several human diseases, especially in cancers including breast cancer, lung cancer, gastric cancer, and CRC (Chen et al., 2020b, c; Gao et al., 2020; Zhang et al., 2020). Indeed, several lines of evidence suggest the involvement of lncRNAs in the development and progression of human CRC, which might in turn serve as novel therapeutic targets (Lan et al., 2018; Tsai et al., 2018; Pichler et al., 2020). It has also been reported that lncRNAs could act as ceRNA to relieve the repressive effect of miRNA on its target genes in CRC (Chen et al., 2017; Xu et al., 2018). However, the role of lncRNAs in CRC, especially in colorectal liver metastasis (CRLM) is largely unknown.

To explore the role of lncRNAs played in CRC and CRLM process, lncRNA microarray using the “LncRNA + mRNA Human Gene Expression Microarray V4.0” was performed and identified a novel CRC-related lncRNA lnc-HSD17B11-1:1. Subsequently, the clinical significance of lnc-HSD17B11-1:1 expression in CRC was explored to gain insights regarding the function and underlying molecular mechanisms of lnc-HSD17B11-1:1 in CRC development and progression. Our data revealed remarkable upregulation of lnc-HSD17B11-1:1 in CRC tissues, association with clinical stage and pathological grade and positive correlation with MACC1 expression. The up-regulation of lnc-HSD17B11-1:1 or MACC1 was closely related to the poor prognosis of patients with CRC. Further functional and mechanistic investigations revealed that lnc-HSD17B11-1:1 could promote cell proliferation and metastasis by acting as a sponge for miR-338-3p in order to relieve micro-RNA repression for MACC1 target gene. Therefore, our data demonstrated that lnc-HSD17B11-1:1 might act as an oncogene in CRC progression, act as a valuable marker and independent prognostic factor for CRC diagnosis, therapy and survival.

## Materials and Methods

### Ethics Statement

This study was approved by local Research Ethics Committee of Peking University Peoples Hospital. Patients who participated in this study were fully informed. After informed, they signed written consents. All the animal experiments involved in this study strictly conform to animal ethics and had approval from the local Research Ethics Committee of Peking University Peoples Hospital, which adheres to generally accepted international guidelines for animal experimentation.

### Patients and Samples

A total of 160 CRC patients who underwent surgery at the Peking University People’s Hospital between 2014 and 2017 were included in this study. Fresh colorectal tumor tissues and matched normal colorectal mucosal tissues were obtained from all the patients. The specimens were obtained and immediately frozen in liquid nitrogen and stored at −80°C until RNA or protein extraction.

### Cell Lines and Cell Culture

The human CRC cell lines SW480, SW620, LoVo, HCT116, HT29, RKO, LS174T, and the normal human colon epithelial cell line NCM460 were purchased from the American Type Culture Collection (Manassas, VA, United States) and were subcultured and preserved. SW480 and SW620 cells were cultured in l-15 medium supplemented with 10% fetal bovine serum (FBS, Gibco) and 100μg/mL streptomycin (Sigma-Aldrich), NCM460, HT-29, HCT116, LS174T and RKO cells were cultured in RPMI1640 medium containing 10% FBS (Gibco) and 100μg/ml streptomycin (Sigma-Aldrich), and LoVo cells were cultured in DMEM medium supplemented with 10% FBS (Gibco) and 100μg/mL streptomycin (Sigma-Aldrich). All these cell lines except SW480 and SW620 (without CO_2_) were maintained at 37°C in a humidified atmosphere containing 5% CO_2_.

### RNA Isolation and Real-Time Quantitative Reverse Transcription Polymerase Chain Reaction (RT-PCR)

Total RNA was extracted with TRIzol reagent (Invitrogen, United States) according to the manufacturer’s instructions. The quality and quantity of isolated RNA were detected by Nanodrop 2000 spectrophotometer (Thermo Fisher Scientific, United States) and the integrity was examined by Agilent 2100 Bioanalyzer (Agilent Technologies, CA, United States). Next, the purified RNA was treated with RiboZero rRNA removal kit (Epicentre, WI, United States) to deplete the rRNA according to the manufacturer’s protocol. The rRNA-depleted RNA samples were randomly fragmented into small pieces and synthesized cDNA with a random primer. The PCR amplification products of cDNA were purified with AMPure XP kit (BeckmanCoulter, CA, United States).

Reverse Transcription Polymerase Chain Reaction was carried out using a One Step SYBR PrimeScript RT-PCR kit (Takara, Dalian, China) and Real-time PCR detection system (Bio-Rad, Hercules, CA, United States) to evaluate the expression of lnc-HSD17B11-1:1. The miRNA miR-338-3p was obtained using the PureLink miRNA isolation kit (Invitrogen), and the quantification of miRNA expression was performed using a TaqMan MicroRNA assay kit (Applied Biosystems, Foster City, CA, United States). The expression of GAPDH genes was assessed simultaneously in all samples as an internal control for lncRNA/mRNA and miRNA expression, respectively. The relative gene expression was determined by 2^–ΔΔ*CT*^ method.

### Quantitative Real-Time PCR (qRT-PCR)

For lncRNA quantification, GAPDH was used as an internal control, and PrimeScriptTM RT MasterMix (TAKARA) was used for reverse transcription and real-time PCR. The primer sequences were as follows: lnc-HSD17B11-1:1 forward: 5′-CTCTGGAACCTGAGGAAGTGG-3′, lnc-HSD17B11-1:1 reverse: 5′-ACGCCTAGTTTTCGGCACTC-3′; MACC1 forward: 5′-CTCTGGAACCTGAGGAAGTGG-3′, lnc-HSD17B11-1:1 reverse: 5′-ACGCCTAGTTTTCGGCACTC-3′; GAPDH forward: 5′-CACCCACTCCTCCACCTTTG-3′, GAPDH reverse: 5′-CCACCACCCTGTTGCTGTAG-3′. All reactions were performed in triplicate. The fold change for each gene relative to the control group was calculated using 2^–ΔΔ*CT*^ method.

### Vector Construction and Cell Transfection

For *in vitro* assays, to interfere the expression of lnc-HSD17B11-1:1, siRNA interference sequences targeting Lnc-HSD17B11-1:1 were designed and synthesized (Ribobio, Guangzhou, China), and a final concentration of 50nM were used for transient transfection. To overexpress lnc-HSD17B11-1:1, full-length human lnc-HSD17B11-1:1 cDNA was cloned into pcDNA3.1 expression vector (Genechem, Shanghai, China). Lipofectamine 3000 (Invitrogen, Carlsbad, CA, United States) was used for transfection according to the manufacturer’s instructions.

For *in vivo* studies, lnc-HSD17B11-1:1 overexpression cell line was used. The lnc-HSD17B11-1:1 gene was cloned into a lentivirus vector LV-GFP-Puro, and SW480 cells were used for infection. Stable transfection of cells was done by puromycin antibiotic selection at a concentration of 2.5 ug/ml for 7 days.

### Cell Proliferation and Colony Formation Assay

HCT116 and SW480 cells (3 × 10^3^ cells) were seeded in 96-well plates in complete medium and infected with lnc-HSD17B11-1:1, mock, si-lnc-HSD17B11-1:1 and si-NC. Cell proliferation assay was conducted with Cell Counting Kit-8 (CCK-8) in accordance with the manufacturer’s instructions. Cells in each group were detected for 6 replicates. Cell proliferation was measured by the CCK-8 method according to the manufacturer’s protocols using a microplate reader (Molecular Devices, Sunnyvale, CA, United States) to measure the absorbance. For colony formation assay, transfected cells were seeded in six-well plate and incubated for 14 days. Then, all the wells were fixed with 4% paraformaldehyde, and stained with 0.1% crystal violet. Image J software was used for statistical analysis of the form colonies.

### Wound Healing, Cell Migration, and Invasion Assays

HCT116 and SW480 cells were seeded in six-well plate. Then, a vertical wound was scratched with a 200 ul microtip in the middle of the wells after 24 h transfection. The width of wounds was measured in three independent positions per group and normalized to control group after 24 h cultured in serum-free medium. Cell invasion assay was carried out using Corning Polycarbonate Membrane insertion transwell chamber (Product #3422, Corning Costar Corp., Cambridge, MA, United States). cells (1 × 10^5^) in serum-free media were placed into the upper chamber while media containing 20% FBS was placed in the lower chamber as a chemoattractant to perform migration assays (without Matrigel) and invasion assays (with Matrigel, Sigma) at 24 h post-transfection. The non-invading cells were erased with cotton swabs after incubation for 48 h. Cells penetrated down to the bottom of the membrane were stained with methanol 0.1% crystal violet. Images were captured by the microscope. The ImageJ software was used for statistical analysis of the number of the cells.

### Nude Mouse Model of Ectopic Tumors

Six-week-old Balb/C nude (nu/nu) mice were purchased from Beijing weitonglihua Experimental Animal Technology Co., Ltd. The tumors were generated by subcutaneously injecting 2 × 10^6^ SW480 infected cells with lnc-HSD17B11-1:1 overexpression cells or control lentivirus particles and suspended in 50L of phosphate buffered saline (PBS) into the dorsal region near the thigh. Four mice were included in each group. The tumor length and width of the mice were measured to assess the tumor size every 7 weeks.

### Lnc-HSD17B11-1:1 Detection BaseScope Assay

BaseScope assays were performed according to the manufacturer’s instructions (Advanced Cell Diagnostics, Newark, CA, United States). The tissues were sectioned into 5-μm thickness, placed onto Superfrost Plus slides (Thermo Fisher Scientific, Loughborough, United Kingdom), and allowed to dry overnight at 25°C. The sections were then baked at 60°C for 1 h before deparaffinizing in xylene (twice for 5 min) and ethanol (twice for 2 min) and dried by baking at 60°C for 2 min. Hydrogen peroxide was applied for 10 min at 25°C, and target retrieval was performed for 15 min at 100°C. This was followed by application of RNAscope^®^ Protease III at 40°C for 30 min, and the samples were rinsed twice in distilled water between treatments. BaseScope probes (Mm-1700024F13Rik, cat# 709881) with positive control (Hs-PPIB, cat # 701031) were then applied and samples were incubated for 2 h at 40°C in a HybEZ oven by the reagents AMP0 (30 min at 40°C), AMP1 (15 min at 40°C), AMP2 (30 min at 40°C), AMP3 (30 min at 40°C), AMP4 (15 min at 40°C), AMP5 (30 min at 25°C), and AMP6 (15 min at 25°C). The slides were rinsed with wash buffer (twice for 2 min) between AMP incubation steps. Finally, the slides were treated with Fast Red for 10 min at 25°C in the dark and counterstained with Gill’s hematoxylin, dried for 15 min at 60°C, and mounted in VectaMount permanent mounting medium (Vector Labs, Burlingame, CA, United States).

### Luciferase Reporter Assay

The sequences of lnc-HSD17B11-1:1 and MACC1 3′UTR and their corresponding mutant versions without miR-338-3p binding sites were synthesized and subcloned into luciferase reporter vector psiCHECK2 (Promega, Madison, WI, United States), and were named as lnc-HSD17B11-1:1-WT, lnc-HSD17B11-1:1-Mut, MACC1 3′UTR-WT and MACC1 3′UTR-Mut, respectively. All these plasmids were validated by sequencing. The relative luciferase activity was examined by dual Luciferase assay kit (Promega, Madison, WI, United States) in accordance with the manufacturer’s protocol.

### RNA Immunoprecipitation (RIP)

RNA Immunoprecipitation was conducted using the Magna RIP kit (Millipore, Billerica, MA, United States) according to the manufacturer’s instructions. SW480 cells were harvested 48 h after transfection of miR-338-3p mimic or miR-NC, and lysed using incomplete RNA lysis buffer. The cell lysates were then incubated with magnetic beads followed by conjugation with anti-Argonaute2 (AGO2) (Millipore, Billerica, MA, United States) or negative control IgG antibody (Millipore, Billerica, MA, United States) at 4°C for 4 h. The beads were washed using washing buffer. The immunoprecipitated RNA and protein were purified and enriched to detect target RNAs and AGO2 by qRT-PCR and western blotting.

### Western Blot Analysis

The total protein of CRC cells was exacted using RIPA buffer, separated by 10% SDS-PAGE, and electrophoresed onto a PVDF membrane (Bio-Rad, CA, United States). The membranes were blocked with 5% skimmed milk powder and incubated with primary antibodies against MACC1 (1:1000) (Abcam, Burlingame, CA, United States), and GAPDH (1:5000) (Cell Signaling Technology, Beverly, MA, United States) at 4°C overnight and then incubated with secondary antibodies (1:5000) (Cell Signaling Technology, Beverly, MA, United States) at room temperature for 2 h. Finally, the bands were examined by ImmobilobTM Western Chemiluminescent HRP Substrate (Millipore, Billerica, MA, United States).

### Statistical Analysis

Statistical analyses were performed by SPSS 22.0 (IBM, SPSS, Chicago, IL, United States) and GraphPad Prism 8.0 (GraphPad Software Inc., CA, United States). The data were presented as means ± standard deviation (SD). The significance between two mean values was assessed using unpaired Student’s *t*-test (two-tailed). The differences between three or more groups were assessed by one-way ANOVA or χ^2^ test. The survival rates were evaluated by Kaplan–Meier method and tested by log-rank test. The effects of clinical variables on the overall survival of CRC patients were determined by univariate and multivariate Cox proportional hazards regression model. In the multivariate Cox proportional hazards regression model, age, gender, T stage, clinical stage and distant metastasis were adjusted for variable analysis. The correlation between groups was analyzed by Pearson correlation. *P*-values of < 0.05 were considered to be statistically significant.

## Results

### LncRNA Expression Profiles in CRC

In order to demonstrate the expression levels of lncRNA in CRC, we previously applied lncRNA microarray and explored the global expression profiles of lncRNAs in CRC primary tissues and liver metastatic tissues (Ye et al., 2016). With a cut-off criteria of fold change >2.0 and *p* < 0.05, a total of 332 distinguishingly expressed lncRNAs were found between CRC liver metastatic tissues and primary tissues, among which 234 were upregulated while 98 were downregulated in liver metastatic tissues. The top 10 upregulated and downregulated lncRNAs were listed in Supplementary Table S1. The lnc-HSD17B11-1:1 (ENST00000508163.1) was shown to be the most upregulated (29.31 folds) lncRNA, which was spliced from lnc-HSD17B11-1, located on chr4:87317169-87345210 and finally formed a circular transcript of 349 nt according to the annotation of lncipedia.^1^

### Elevated lnc-HSD17B11-1:1 Is Expressed in Colorectal Cancer Tissues and Is Correspond With a Poor Prognosis in Colorectal Cancer Patients

**FIGURE 1:**
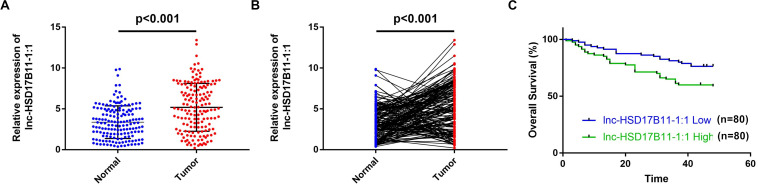
lnc-HSD17B11-1:1 is upregulated and indicated poor prognosis in CRC. **(A,B)** qRT-PCR analysis of lnc-HSD17B11-1:1 expression in CRC tissues (Tumor) and corresponding normal colorectal mucosa tissues (Normal) (*n* = 160). **(C)** Kaplan–Meier survival curve of overall survival in 160 patients with CRC on the basis of the lnc-HSD17B11-1:1 expression. Patients were divided into high and low group by median expression.

In accordance with the microarray data, the results revealed that lnc-HSD17B11-1:1 was markedly upregulated in CRC tissues and cell lines when compared with adjacent non-tumor tissues and normal colon cell lines (Figures 1A,B). The relationship between lnc-HSD17B11-1:1 expression and clinical characteristics of the CRC patients were listed in Table 1. The expression of lnc-HSD17B11-1:1 showed significant correlation with T stage (*p* = 0.043), clinical stage (*p* = 0.027), and distant metastasis (*p* = 0.003). Kaplan–Meier survival curve indicated that the expression levels of lnc-HSD17B11-1:1 were inversely correlated with the overall survival of CRC patients (Figure 1C). Further univariate and multivariate Cox regression analysis revealed that lnc-HSD17B11-1:1 expression level were independent prognostic factors for patients with CRC (Table 2).

**TABLE 1:** Association of lnc-HSD17B11-1:1 expression and clinicopathological features in CRC patients.

Clinicopathological features	Expression of Lnc-HSD17B11-1:1		*P*-value
	Low	High	
Gender			0.527
Male	44	40	
Female	36	40	
Age at diagnosis			0.504
≤60	29	25	
>60	51	55	
Tumor size (cm)			0.264
≤5	42	37	
>5	38	43	
Location			0.391
Proximal	27	22	
Distal	53	58	
Differentiation			0.429
Well-moderate	14	18	
Poor	66	62	
Cancer nodules			0.428
No	35	40	
Yes	45	40	
Vessel carcinoma embolus			0.262
No	48	43	
Yes	32	37	
Depth of invasion			0.043*
T1–T2	16	7	
T3–T4	64	73	
Lymph node metastasis			0.564
No	48	48	
Yes	32	32	
TNM stage			0.027*
I–II	12	3	
III–IV	68	77	
distant metastasis			0.003*
No	74	60	
Yes	6	20	

*p < 0.05.

**TABLE 2:** Univariate and multivariate Cox regression analysis of lnc-HSD17B11-1:1 and survival in patients with CRC.

Clinical variables	Univariate analysis		*P*	Multivariate analysis		*P*
	HR	95%Cl		HR	95%Cl	
Age (≥50 vs. <50)	0.952	0.415–1.921	0.814			
T stage (T1/T2 vs. T3/4)	1.722	1.012–3.021	0.046*			
N stage (N0 vs. Nx)	0.853	0.442–1.714	0.725			
TNM stage (I-II vs. III-IV)	2.296	1.141–4.617	0.030*	3.092	1.221–6.102	0.025*
lnc-HSD17B11-1:1 (low vs. high)	4.652	2.866-15.108	0.012*	5.042	2.604–16.105	<0.001*

HR hazard ratio, CI confidence interval. *P < 0.05.

### Lnc-HSD17B11-1:1 Regulates Proliferation, Tumor Growth, Migration and Invasion in Colorectal Cancer Cells

**FIGURE 2:**
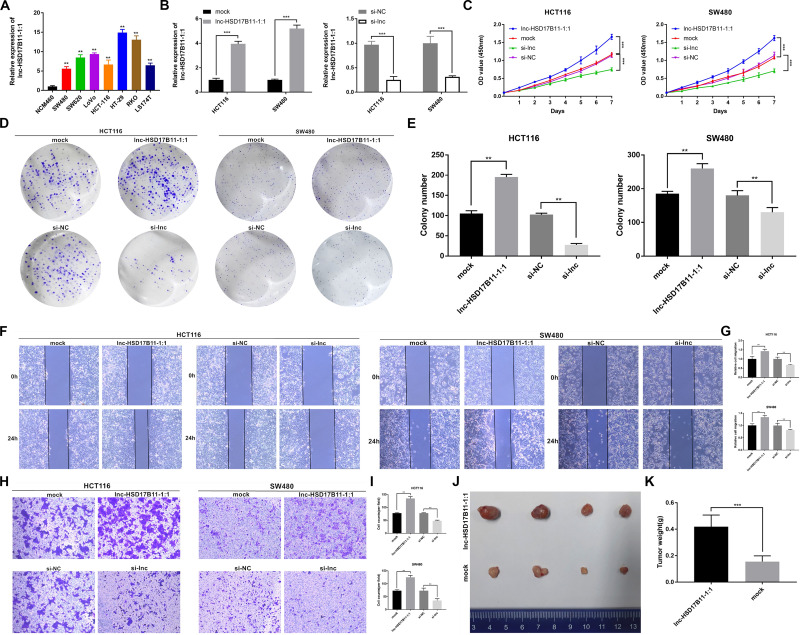
lnc-HSD17B11-1:1 promotes cell proliferation, tumor growth, migration and invasion *in vitro* and *in vivo* in CRC. **(A)** Relative expression of lnc-HSD17B11-1:1 in CRC and NCM460 cell lines was examined by qRT-PCR. **(B)** Relative expression of lnc-HSD17B11-1:1 in HCT116 and SW480 cells transfected with lnc-HSD17B11-1:1 expression vector, mock, sh-lnc or sh-NC, respectively. **(C)** Growth curves of cells of various groups were evaluated by CCK-8 assays. **(D,E)** Colony formation assays were detected by proliferation of cells transfected with indicated vectors. **(F,G)** Cell migration capacities were conducted by wound healing assays after transfection (magnification, ×50). Scale bar, 200μm. **(H,I)** Cell invasion abilities were indicated by transwell assays in each group (magnification, ×100). Scale bar, 100 μm. **(J)** Images of subcutaneous xenotransplantation of tumors in each group was photographed (*n* = 4). **(K)** The weight of each group was displayed. Data were showed as mean ± SD, **P < 0.01, ***P < 0.001, N.S, non-significant.

To investigate the biological function of lnc-HSD17B11-1:1 in CRC cells, we first detected lnc-HSD17B11-1:1 expression levels in CRC cell lines and found high expression in cancer cell lines in comparison with colorectal normal one NCM460, especially in HCT116 and SW480 (Figure 2A). In consequence, these two cell lines were chosen for further study. Lnc-HSD17B11-1:1 was overexpressed or down-regulated in HCT116 and SW480 cells which were transfected with overexpression or siRNA vector independently by qRT-PCR (Figure 2B). CCK-8 assays were applied to demonstrate that upregulation of lnc-HSD17B11-1:1 could significantly promote cell growth of HCT116 and SW480 cells, while downregulation of lnc-HSD17B11-1:1 markedly suppressed the proliferation viability (Figure 2C). Then colony formation assays further displayed that the upregulation of lnc-HSD17B11-1:1 could markedly enhance the cell cloning capabilities of HCT116 and SW480 whereas downregulation of lnc-HSD17B11-1:1 significantly reduces the cell cloning capabilities (Figures 2D,E). Collectively, all these data together indicated that lnc-HSD17B11-1:1 facilitates proliferation and tumor growth of CRC cells. Then, wound healing and transwell assays were performed to examine the effects of lnc-HSD17B11-1:1 on migration and invasion of CRC cells. The results suggested that the migration and invasion abilities of HCT116 and SW480 cells were strongly strengthened by upregulation of lnc-HSD17B11-1:1 and notably suppressed by downregulation of lnc-HSD17B11-1:1 (Figures 2F–I).

### lnc-HSD17B11-1:1 Promotes Tumorigenesis and Metastasis of CRC Cells *in vivo*

To determine the effects of lnc-HSD17B11-1:1 on tumor growth and liver metastasis *in vivo*, SW480 cells were stably transfected with overexpression or mock vector and infected with LV-NC or LV-circ and then were subcutaneously injected into female nude mice. As a result, the tumors derived from cells that overexpress lnc-HSD17B11-1:1 were bigger and heavier when compared to in the control group (Figures 2J,K). These results furthermore verified the function of promoting tumor growth of lnc-HSD17B11-1:1 in CRC, suggesting that lnc-HSD17B11-1:1 might be an oncogene and play a vital role in the progress colorectal cancer.

### lnc-HSD17B11-1:1 Functions as a Sponge for miR-338-3p

**FIGURE 3:**
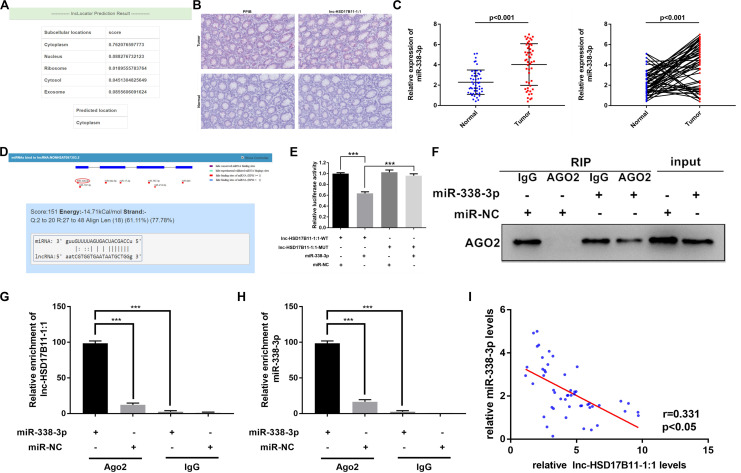
lnc-HSD17B11-1:1 acts as a sponge for miR-338-3p. **(A)** The location of lnc-HSD17B11-1:1was predicted in the cytoplasm on the website of lncLocator. **(B,C)** Base Scope Assay was conducted to identify the subcellular location of lnc-HSD17B11-1:1 (red) in CRC tissues (magnification, ×20, scale bar, 100 μm). **(C)** qRT-PCR assay of miR-338-3p expression in CRC tissues (Tumor) and corresponding normal colorectal mucosa tissues (Normal) (*n* = 50). **(D)** The miR-338-3p binding site on lnc-HSD17B11-1:1 predicted by lncRNASNP. **(E)** The relative luciferase activities were conducted in SW480 cells after transfection with miR-338-3p mimics or miR-NC and lnc-HSD17B11-1:1-WT or lnc-HSD17B11-1:1-MUT, respectively. **(F,G)** RIP assay was performed in SW480 cells transfected with miR-338-3p mimic or miR-NC. qRT-PCR and western blot were applied to detect AGO2 protein, lnc-HSD17B11-1:1 and miR-338-3p, respectively. **(H)** qRT-PCR assay of relative miR-338-3p expression was conducted after transfection with indicated vectors. **(I)** Pearson correlation analysis of lnc-HSD17B11-1:1 and miR-338-3p expression was analyzed in 50 CRC cohorts. Data were indicated as mean ± SD, ***P < 0.001.

Considering that lncRNAs could cat as miRNA sponges in cytoplasm, we firstly predicted the sublocalization in cell of lnc-HSD17B11-1:1on the website of lncLocator,^2^ and the results predicted that lnc-HSD17B11-1:1 mainly located in cytoplasm (Figure 3A). Base Scope assay was performed to observe the subcellular localization of lnc-HSD17B11-1:1 with PPIB control in CRC tumor tissues and adjacent normal tissues. Indeed, the results are in coincidence with predicted that most of lnc-HSD17B11-1:1 (red) were located in cytoplasm (Figure 3B). Then, the levels of miR-338-3p were evaluated in 50 pairs of CRC tumor tissues and corresponding normal colorectal mucosa tissues. The results revealed that miR-338-3p was significantly downregulated in CRC tumor tissues (Figure 3C).

Then we predicted the potential conserved targets of lnc-HSD17B11-1:1 binding on miRNA according to lncRNASNP2^3^ to clarify the molecular mechanism underlying lnc-HSD17B11-1:1. The results indicated that lnc-HSD17B11-1:1 possesses target sequence of miR-338-3p with a high score (Figure 3D). Therefore, we hypothesized that lnc-HSD17B11-1:1 might serve as a ceRNA for miR-338-3p. In order to verify the bioinformatics prediction analysis on the website, dual-luciferase reporter assay was performed in SW480 cells. And the results revealed that the activity of luciferase reporter vector with lnc-HSD17B11-1:1-WT sequence could be significantly decreased by miR-338-3p mimics compared with control groups (Figure 3E), indicating that there might be a direct interaction between lnc-HSD17B11-1:1 and miR-338-3p. Then RIP assay was performed in SW480 cells to pull down anti-AGO2 antibody along with the RNA transcripts binding to AGO2, and IgG as a negative control. As a result, miR-338-3p and lnc-HSD17B11-1:1 were all availably pulled down by anti-AGO2 antibodies compared with IgG, and both lnc-HSD17B11-1:1 and miR-338-3p were significantly concentrated in cells transfected with miR-338-3p mimics compared with miR-NC group (Figures 3F–H). Then Pearson correlation analysis revealed a prominently negative correlation between the expression of lnc-HSD17B11-1:1 and miR-338-3p in 50 CRC patients (Figure 3I). These results demonstrated that lnc-HSD17B11-1:1 may act as a sponge for miR-338-3p in CRC.

### MACC1 Is Directly Targeted by miR-338-3p and Indirectly Regulated by lnc-HSD17B11-1:1

**FIGURE 4:**
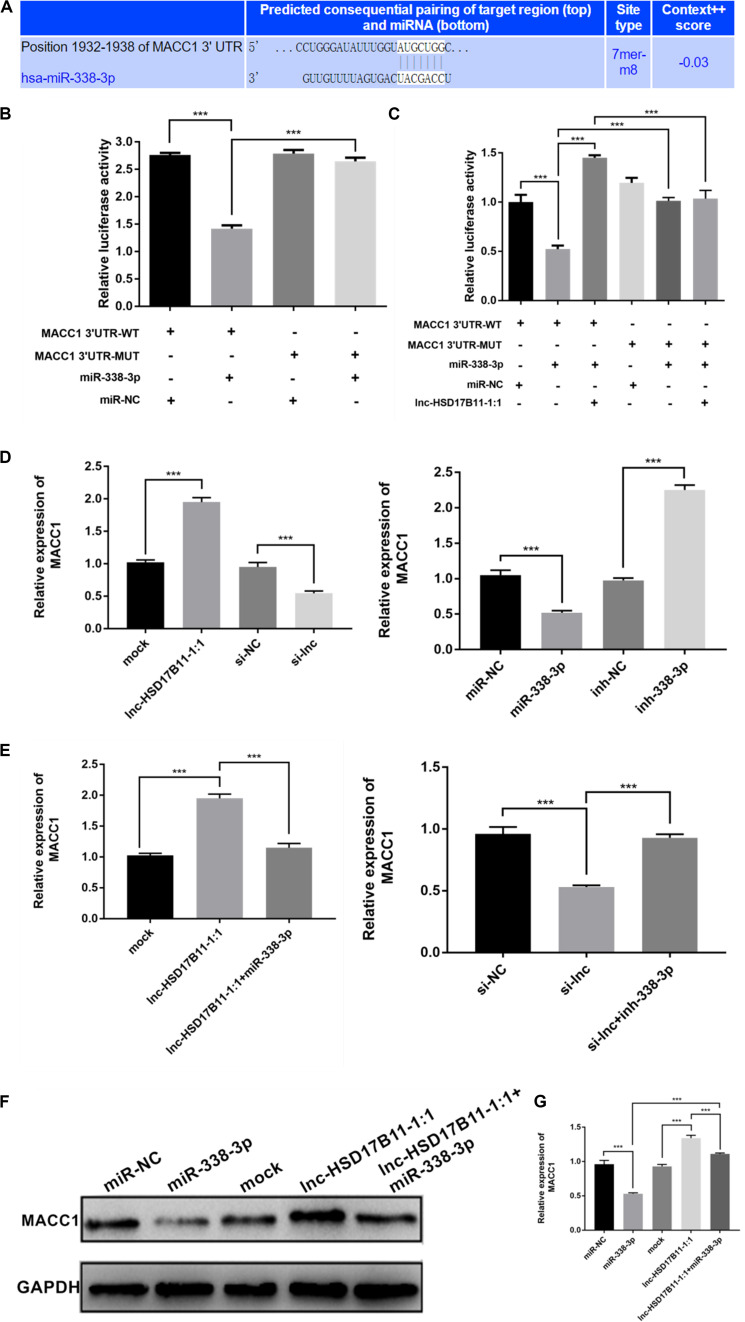
MACC1 is directly regulated by miR-338-3p and indirectly influenced by lnc-HSD17B11-1:1. **(A)** The miR-338-3p binding site on MACC1 was predicted by targetScan. **(B,C)** The relative luciferase activities were detected in SW480 cells transfected with miR-338-3p mimics or miR-NC and MACC1 3′UTR-WT or MACC1 3′UTR-MUT, and lnc-HSD17B11-1:1, respectively. **(D)** qRT-PCR assay of relative expression of MACC1 were conducted in SW480 cells transfected with miR-NC, miR-338-3p, ihn-NC, and inh-338-3p, respectively. **(E)** qRT-PCR assay of relative expression of MACC1 was tested in cells after transfection with indicated miRNAs, inhibitors or vectors. **(F,G)** Western blot assay were performed to detect the relative expression of MACC1 protein level in SW480 cells transfected with indicated inhibitors, vectors or miRNAs. Data were indicated as mean ± SD, ***P < 0.001.

According to TargetScan,^4^ lnc-HSD17B11-1:1 and MACC1 share the same miRNA response element (MRE) of miR-338-3p. Dual luciferase reporter assay were performed to validate the prediction. As expected, the results showed that miR-338-3p mimics could significantly decrease the luciferase activity of MACC 1 3′UTR-WT group compared with control groups (Figures 4A,B). Moreover, the luciferase activity was recovered after transfection with lnc-HSD17B11-1:1 in the miR-338-3p + MACC1-3′UTR-WT group while MACC1-3′UTR-MUT abrogated the suppressive effect (Figure 4C).

Additionally, we found that overexpression of lnc-HSD17B11-1:1 or downregulation of miR-338-3p dramatically increased the levels of MACC1 while knockdown of lnc-HSD17B11-1:1 or upregulation of miR-338-3p remarkedly decreased the expressions of MACC1 (Figure 4D). Moreover, the increase or decrease of MACC 1 caused by lnc-HSD17B11-1:1 overexpression or knockdown could be reversed by miR-338-3p mimics or inhibitors, respectively (Figure 4E). Western blot demonstrated that upregulation of lnc-HSD17B11-1:1 and downregulation of miR-338-3p increased the protein levels of MACC1 respectively, and the effects caused by overexpression of lnc-HSD17B11-1:1 could be reversed by miR-338-3p mimics vice versa (Figures 4F,G). These data suggest that lnc-HSD17B11-1:1 could regulate the expression of MACC 1 through serving as a ceRNA for miR-338-3p in CRC.

### The Effect of MACC1 Expression on the Survival of CRC Patients and Its Correlation With lnc-HSD17B11-1:1 in CRC Specimens and in Nude Mice Samples

**FIGURE 5:**
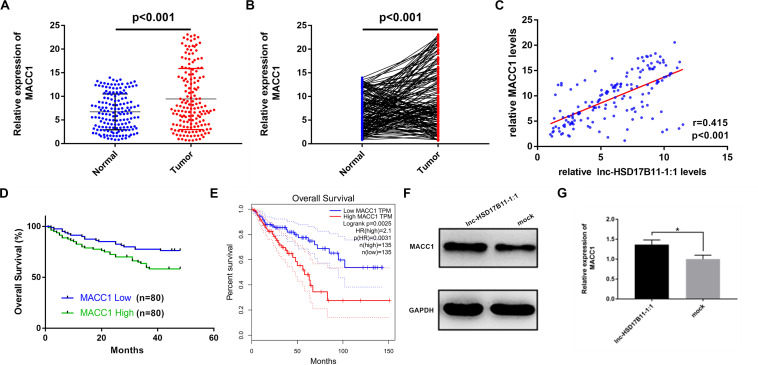
The effect of MACC1 expression on the survival of CRC patients and its correlation with lnc-HSD17B11-1:1 in CRC specimens and nude mice samples. **(A,B)** Relative expression of MACC1 in CRC tissues (Tumor) and compared with normal tissues (Normal) was detected by qRT-PCR (*n* = 160). **(C)** Pearson correlation analysis of lnc-HSD17B11-1:1 and MACC1 expression was performed in 160 CRC cohorts. **(D)** Kaplan–Meier survival curve of overall survival in 160 patients with CRC according to the MACC1 expression. Patients were stratified into high and low expression group by median expression. **(E)** Relative expression of MACC1 in CRC tissues (Tumor) corresponding normal colorectal mucosa tissues (Normal) was analyzed using TCGA data. Kaplan–Meier survival analysis of overall survival based on TCGA data (*n* = 270). **(F,G)** Western Blot was applied to determine the protein level of MACC1 in two groups of nude mice tumor samples. Data were indicated as mean ± SD, *P < 0.05.

To ensure whether MACC1 is co-overexpressed with lnc-HSD17B11-1:1 in CRC patients, the levels of MACC1 were examined in the 160 pairs of CRC tissues and para-cancerous tissues by qRT-PCR. The results demonstrated that MACC1 was considerably upregulated in CRC (Figures 5A,B). Pearson correlation analysis suggested that the expression levels of lnc-HSD17B11-1:1 were positively associated with those of the MACC1 in 160 CRC patients (Figure 5C). Kaplan–Meier survival analysis based on our own data revealed that the higher level of MACC1 was related with poor prognosis (Figure 5D), while the TCGA data also showed that the higher level of MACC1 was correlated with poor prognosis (Figure 5E). These data validated that the synergic action of lnc-HSD17B11-1:1 and MACC1 might participate in the tumorigenesis and progress of CRC. In addition, in nude mice samples, Western blot also revealed that the expression of MACC1 in lnc-HSD17B11-1:1 overexpressed group was much higher than that in mock group (Figures 5F,G). In summary, these data suggest that lnc-HSD17B11-1:1 could promote the expression of MACC1 acting as an oncogenic gene and forecast poor prognosis of patients with CRC.

## Discussion

Some lncRNAs were reported to function as oncogenes or tumor suppressors in different types of cancers including CRC (Jin et al., 2020; Shang et al., 2020; Shen et al., 2020; Wang et al., 2020; Wei et al., 2020; Zheng et al., 2020). only few lncRNAs in CRC have been functionally characterized and the biological function of most of the lncRNAs remains largely unknown. So, lncRNA microarray chip was performed to obtain the expression profiles of lncRNA in three pairs of CRLM tissues and primary tumor tissues. Subsequently, a novel lncRNA-lnc-HSD17B11-1:1 was identified, which showed obvious upregulation in CRC tissues and was significantly correlated with T stage, clinical stage, distant metastasis as well as poor overall survival in patients with CRC. Furthermore, functional experiments both *in vivo* and *in vitro* demonstrated that lnc-HSD17B11-1:1 significantly promoted proliferation and metastatic abilities of CRC cells, while knockdown of lnc-HSD17B11-1:1 showed an inverse effect.

A growing body of research suggests that some lncRNAs could serve as sponges for miRNAs to regulate the expression of target mRNA in CRC (Chen et al., 2020a). For instance, LINC00461 may be an oncogene in CRC by targeting miR-323b-3p through NFIB signaling pathway (Yu et al., 2019), Moreover, lncRNA MIR4435-2HG was reported to play an oncogenic role by promoting CRC growth and metastasis and establish the presence of MIR4435-2HG/miR-206/YAP1 axis in CRC (Dong et al., 2020). Besides, LINC02595 is a prognostic marker through LINC02595/miR-203b/BCL2L1 axis in CRC (Yang et al., 2020). In our research, we found lnc-HSD17B11-1:1 comprised the MRE of miR-338-3p through bioinformatics analyses. Then bioinformatics analyses and Base Scope assay both revealed that lnc-HSD17B11-1:1 was located in cytoplasm of CRC tumor tissues. Hence, we speculated that lnc-HSD17B11-1:1 might be serves as an oncogene by sponging miR-338-3p in CRC. qRT-PCR, Dual-luciferase reporter and anti-AGO2 RIP assays were conducted to confirm that lnc-HSD17B11-1:1 could interact with miR-338-3p directly. Furthermore, we detected that miR-338-3p was markedly downregulated in CRC tissues. In consistence with our research, miR-338-3p was reported prominently decreased in CRC tumor tissues and positively correlated with the prognosis of CRC (Yang et al., 2020). Another research demonstrated that miR-338-3p inhibited CRC proliferation, invasion and metastasis by targeting MACC1 (Zou et al., 2018). Moreover, miR-338-3p is also reported to act as a tumor suppressor for sorts of other malignancies, such as lung and liver cancer (Sun et al., 2013, 2015; Wang et al., 2015). In summary, our results demonstrated the significance of lnc-HSD17B11-1:1 in tumor genesis and progress of CRC and revealed that lnc-HSD17B11-1:1 play an oncogenic role via sponging miR-338-3p.

MACC1 is a vital gene with the invasive behaviors and postoperative liver metastasis in colon cancer (Stein et al., 2009). We found lnc-HSD17B11-1:1 and MACC1 are co-overexpressed in CRC tumor tissues. Then, bioinformatics analysis revealed that MACC1 is an underlying target of miR-338-3p by TargetScan. Dual-luciferase reporter assay were conducted to validate that miR-338-3p could directly target MACC1-3′UTR and suppress its expression. Furthermore, the suppressive effect by miR-338-3p could recovered by overexpression of lnc-HSD17B11-1:1. It has also been reported that overexpression of MACC1 was connected with poor prognosis in diverse of cancers (Lin et al., 2020; Makino et al., 2020). According to a retrospective cohort study, MACC1 is differentially expressed in CRC and predicts best aggressive clinicopathological features, tumor budding, metastasis formation and poor survival outcome (Koelzer et al., 2015). In consistence with previous studies, we revealed that MACC1 was remarkably overexpressed in CRC tissues and upregulation of MACC1 was in coincidence with poor survival. To confirm the relationship between lnc-HSD17B11-1:1 and MACC1, we conducted qRT-PCR and western blot assays to identify that upregulation of lnc-HSD17B11-1:1 could promote the expression of MACC1 at both mRNA and protein levels, vice versa. while the regulation effects could be partially reversed by miR-338-3p mimics or inhibitors, respectively, which notably support our hypothesis that lnc-HSD17B11-1:1 act as a ceRNA to promote MACC1-mediated proliferation and metastasis via decoying miR-338-3p in CRC.

Taken together, our results demonstrate that lnc-HSD17B11-1:1 plays an important role in the progression of human CRC by functioning as a ceRNA to regulate the expression of MACC1 through miR-338-3p sponge activity. The pleiotropic effects of lnc-HSD17B11-1:1 on the pathogenesis of CRC suggests that it has the potential to act as a potential target for CRC therapy.

## Data Availability Statement

The raw data supporting the conclusions of this article will be made available by the authors, without undue reservation, to any qualified researcher.

## Ethics Statement

The studies involving human participants were reviewed and approved by the Research Ethics Committee of Peking University Peoples Hospital. The patients/participants provided their written informed consent to participate in this study. The animal study was reviewed and approved by the Research Ethics Committee of Peking University People’s Hospital.

## Author Contributions

WZ, BW, and SW conceived the study and participated in its design and coordination. WZ drafted and revised the manuscript. KJ and ZS collected the clinical data and helped with the statistical analysis. ZZ and QW performed the experiments. SW conceived the study and revised the manuscript. All authors read and approved the final manuscript.

## Conflict of Interest

The authors declare that the research was conducted in the absence of any commercial or financial relationships that could be construed as a potential conflict of interest.

## Footnotes

1 https://lncipedia.org/

2 http://www.csbio.sjtu.edu.cn/bioinf/lncLocator

3 http://bioinfo.life.hust.edu.cn/lncRNASNP

4 http://www.targetscan.org/vert_72

## Supplementary Material

TABLE S1 The top 10 upregulated and downregulated lncRNAs.
