# Supplementary material for: Lnc-HSD17B11-1:1 Functions as a Competing Endogenous RNA to Promote Colorectal Cancer Progression by Sponging miR-338-3p to Upregulate MACC1
Source: Front Genet. 2020 Jun 12;11:628. doi: 10.3389/fgene.2020.00628 (PMC7304498; doi:10.3389/fgene.2020.00628)
Supplement: TABLE S1 — The top 10 upregulated and downregulated lncRNAs. [file Table_1.docx]

The top 10 upregulated and downregulated lncRNAs

| Upregulated lncRNAs | | | Downregulated lncRNAs | | |
| --- | --- | --- | --- | --- | --- |
| lncRNA ID | Gene Name | Fold change | lncRNA ID | Gene Name | Fold change |
| ENST00000508163.1 | ENSG00000255723.1 | 39.31835 | lnc-GNAT1-1 |  | 42.818486 |
| ENST00000417851.1 | ENSG00000188338.10 | 28.74565 | TCONS_00007196 | XLOC_003181 | 35.274246 |
| ENST00000561529.1 | ENSG00000260886.1 | 21.4391 | TCONS_00005201 | XLOC_002209 | 27.245037 |
| TCONS_00022351 | ENSG00000258867.1 | 18.56326 | TCONS_00007197 | XLOC_003181 | 22.070576 |
| ENCODE_44_3620 | ENSG00000232224.1 | 16.80691 | RNA143581 |  | 12.06795 |
| ENST00000589927.1 | ENSG00000186526.7 | 16.63199 | ENST00000591929.1 | ENSG00000235790.2 | 12.061668 |
| NR_024424.1 | XLOC_004271 | 13.96551 | TCONS_00025507 | XLOC_012317 | 12.056485 |
| ENCODE_2546_372 | ENSG00000233396.1 | 13.95641 | RNA143595 |  | 12.035239 |
| ENST00000427428.2 | ENSG00000188338.10 | 13.31409 | RNA143580 |  | 12.025375 |
| HIT000090682 | ENSG00000215447.3 | 12.60362 | HIT000085899 | HIX0080295 | 12.020649 |
